# The complete chloroplast genome of *Sesbania cannabina*

**DOI:** 10.1080/23802359.2019.1662754

**Published:** 2019-09-10

**Authors:** Wei Tan, Han Gao, Xiaolei Yu, Huanyu Zhang, Weiling Jiang, Xiaoxuan Tian

**Affiliations:** Tianjin State Key Laboratory of Modern Chinese Medicine, Tianjin University of Traditional Chinese Medicine, Tianjin, China

**Keywords:** *Sesbania cannabina*, chloroplast, genome

## Abstract

*Sesbania cannabina* is an annual plant widely distributed in south China, southeast Asia, and Australia. In the present study, we sequenced the complete chloroplast genome of *S. cannabina,* which is 153,978 bp in length and it is characterized by a typical quadripartite structure composed of a LSC (85,406 bp) and SSC (19,112 bp) region interspersed by a pair of 23,730 bp IR regions. It encodes 125 genes, comprising 81 protein-coding genes, 8 rRNA genes, and 36 tRNA genes. Phylogenetic analysis confirmed the position of *S. cannabina* within the subfamily Papilionoideae.

*Sesbania*, a genus of flowering plants in Papilionoideae subfamily, is the only genus of the tribe Sesbanieae, consisting of approximately 20 species. Moreover, *Sesbania* is increasingly used as a green manure crop in rice growing systems (Becker et al. 1990). Among these species, *Sesbania cannabina* is one of the most common species.

Previous studies have demonstrated that *S. cannabina* can promote the drainage of paddy-upland rotated and ill-drained fields (Itoh et al. 1992). However, no complete chloroplast (cp) genome of *S. cannabina* has been released. Therefore, in the present study, we determine the complete cp genome sequence of *S. cannabina* as well as the position of *S. cannabina* within the subfamily Papilionoideae.

The seed of *S. cannabina* was purchased from Anguo, Hebei, China (115.32164°E, 38.41844°N). The voucher specimens (voucher number: TJZ-1) were deposited at Tianjin State Key Laboratory of Modern Chinese Medicine. DNA isolation was carried out according to the standard protocol of Extract Genomic DNA Kit. Afterward, the resulting DNA was sequenced using an Illumina HiSeq platform. The paired-end reads were assembled to contigs using NOVOPlasty v3.1 (Dierckxsens et al. 2017) and GetOrganelle pipeline (Jin et al. 2018) with default settings. Then, the total contigs were circularized by mapping to reference (KJ468102). The cp genome of *S. cannabina* was annotated using GeSeq (Tillich et al. 2017) and CpGAVAS2 (Shi et al. 2019), inspected and adjusted manually. The online tRNAscan-SE service was used to verify tRNA genes of the *S. cannabina.* The circular cp genome map was drawn by OGDRAW (Lohse et al. 2013). The final complete cp genome was deposited into GenBank with accession number MN105118.

The chloroplast genome of *S. cannabina* is 153,978 bp in length, including two short-inverted repeats (IRa and IRb) of 23,730 bp, divided into SSC (19,112 bp) and LSC (85,406 bp). In addition, a total of 125 genes, containing 81 protein-coding genes, 8 rRNA genes, and 36 tRNA genes are identified.

To resolve the phylogenetic position of *S. cannabina* within the subfamily Papilionoideae, 35 Papilionoideae species plastomes were obtained from Genbank database for phylogenetic analysis. The complete chloroplast genome sequences were aligned using MAFFT version 7 (Katoh and Standley 2013). Maximum likelihood (ML) analysis was performed with RAxML v8 (Stamatakis 2014) based on the GTRGAMMAI model using 1000 replicates of bootstrap analysis. The results (Figure 1) illustrated that *S. cannabina* was closest to *Robinia pseudoacacia*. The results of this study will facilitate the species identification, molecular biology, and phylogenetic studies of *S. cannabina*.

**Figure 1.**
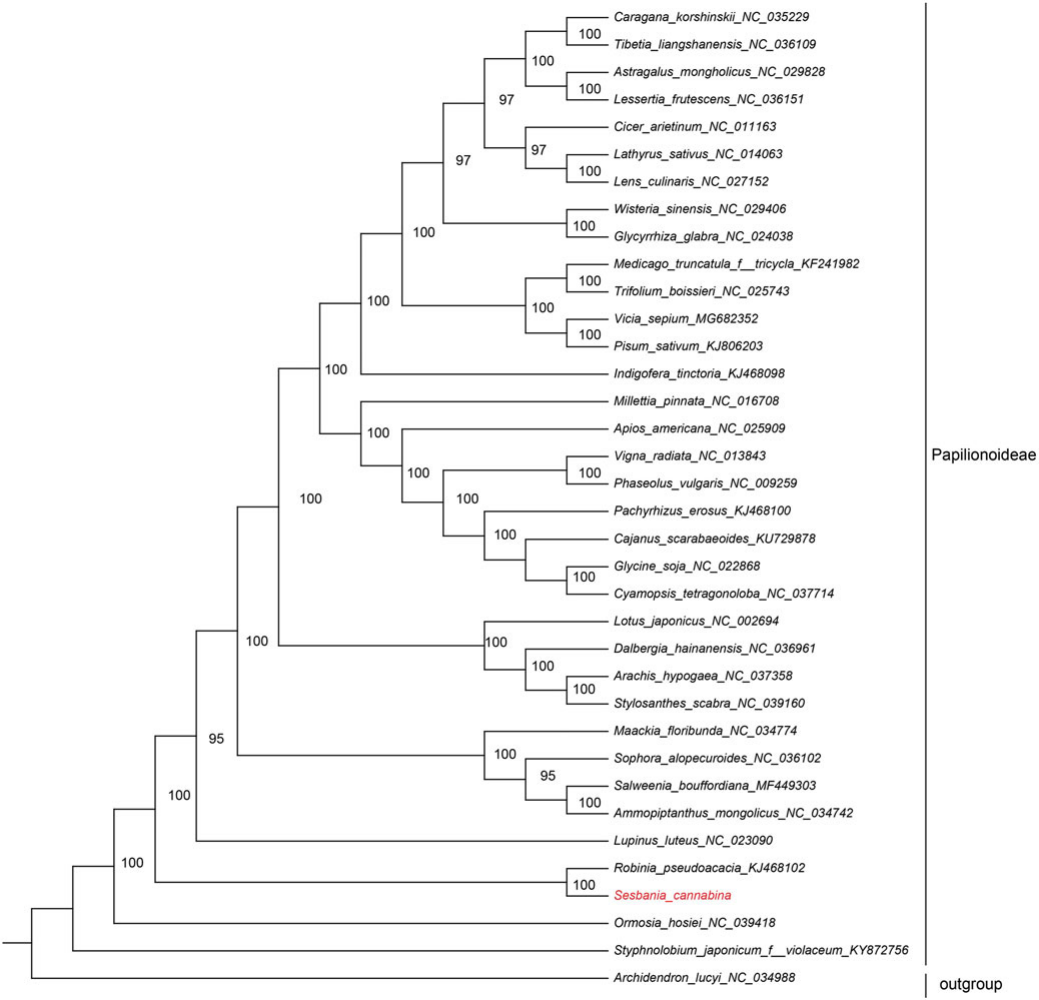
ML phylogenetic tree of Papilionoideae with 36 species was constructed by complete chloroplast genome sequences. Numbers on the nodes are bootstrap values from 1000 replicates. *Archidendron lucyi* was selected as outgroup.
